# PReS-FINAL-2275: Improvement of calcinosis cutis with intravenous pamidronate in a 2-year-old girl with progressive widespread skin calcification of unknown origin

**DOI:** 10.1186/1546-0096-11-S2-P265

**Published:** 2013-12-05

**Authors:** S Mrusek, A Jeibmann, E Lausch, G Ganser

**Affiliations:** 1 Department of Pediatric Rheumatology, Northwest German Center of Rheumatology, St. Josef Stift Sendenhorst, Sendenhorst, Germany; 2 Institute of Neuropathology, University Hospital Münster, Münster, Germany; 3 Pediatric Genetics, University Hospital Freiburg, Freiburg, Germany

## Introduction

Pathologic calcification occurs in up to 40% of children with juvenile dermatomyositis and can develop within several months even in successfully treated patients. Other differential diagnoses of calcinosis cutis in children are rare. There is no generally accepted standard treatment of calcinosis. We report on a 2-year-old previously healthy girl, the 2^nd ^child of non-consanguineous healthy parents from Kosovo, who presented with a 7-month history of progressive calcinosis cutis primarily affecting both legs. The girl had no other signs or symptoms of juvenile dermatomyositis or other autoimmune diseases.

## Objectives

An extensive diagnostic work-up was carried out to find an underlying cause and an effective treatment.

## Methods

Laboratory studies, immunology tests, X-rays, magnetic resonance imaging and skin and muscle biopsy were done.

## Results

Blood tests showed mild leukocytosis (14.0 × 10^9^/L), lymphocytosis (9.1 × 10^9^/L) and thrombocytosis (632 × 10^9^/L), hypergammaglobulinemia (1786 mg/dl) and elevated S100A8/S100A9 protein levels (2230 ng/ml). Significantly elevated plasma osteopontin levels (>30-fold) were noted. Creatinkinase, aldolase, C-reactive protein, erythrocyte sedimentation rate, liver function tests, electrolytes, vitamin D and parathormone levels were all normal. ANA, ENA, rheumatoid factor, myositis-specific and associated antibodies (anti-Mi-2, anti-Jo-1, anti-Pm/Scl, anti-SRP54), complement C3 and C4 were all negative. X-rays of her legs showed extensive calcinosis. On magnetic resonance imaging, there was contrast enhancement of thickened muscle fascia but no signs of dermatomyositis. Skin and muscle biopsy revealed calcification (without ossification!) with foreign body reaction in subcutaneous fatty tissue and inflammatory infiltrates (lymphocytes and macrophages), but no signs of eosinophilic fasciitis, neurogenic or myopathic damage. We suspected an autoimmune disease with fasciitis (primary or secondary?) and calcinosis cutis and started immunosuppressive therapy with prednisone and azathioprine. After calcinosis progressed (clinically, on x-rays, ultrasound and follow-up MRI), methotrexate was added and pamidronate infusions were started. Three months later, improvement of calcinosis and local tissue inflammation as well as no development of new calcium deposits were observed. Two further cycles of pamidronate every three to four months are planned and a slow tapering of the immunosuppressive therapy.

## Conclusion

The underlying cause of the disease in our patient remains unclear. The degree and clinical distribution of the calcinosis, the young age of our patient as well as the very high levels of osteopontin, a major regulatory protein of calcification, suggest an autoimmune disease. The patient's differential diagnosis includes an unknown genetic disease of calcium metabolism - we are still awaiting the results of a whole exome analysis of the patient and her family members. Immunosuppressive therapy did not stop progression of calcinosis and only after starting pamidronate rapid regression of calcified lesions was noted. Whether this therapy has any long-term beneficial effect and whether the immunosuppressive therapy can be fully tapered remains to be seen.

## Disclosure of interest

None declared.

